# Post-Graduate School

**Published:** 1889-05

**Authors:** 


### Post-Graduate School.

It is with great satisfaction that we make mention of the establishment of an institution for instruction in prosthetic dentistry, and that too under the direction of one of the most proficient practitioners in this department in this or any other country, viz., Dr. L. P. Haskell, of Chicago. He is acknowledged as authority in prosthetic dentistry. Having followed this department with untiring perseverance and industry for more than thirty years, and in addition to this he has brought into use every available facility and appliance for securing the highest results; and these all have been operated with the most perfect system.

The object in the establishment of this department is to afford to practitioners and recent graduates from our colleges an opportunity to carry their work in this line to a higher point of perfection than it has been possible for them to do under the circumstances in which they were placed. Of the importance and value of such a department there can be no question. And four-fifths of the practitioners of the country would be benefited by the advantages here offered, of which many could easily avail themselves, as a long course is not exacted nor is it expensive. In these respects it is brought within the reach of almost every one.

Dr. M. Stout is Secretary to whom all letters of inquiry should be addressed. The announcement, the main points of which is found in the advertising pages of this number will be sent to any one who apply.

